# Neutrophils - a complex story in the pathogenesis of systemic lupus erythematosus

**DOI:** 10.1186/ar4640

**Published:** 2014-09-18

**Authors:** Christian Lood, Keith Elkon

**Affiliations:** 1 University of Washington, Seattle, WA, USA

## Background

Neutrophils have long been recognized as important contributors of inflammation and tissue destruction in systemic lupus erythematosus (SLE). The LE cell (engulfment of antibody-opsonized nuclear material by neutrophils) was for decades regarded as the hallmark of SLE. With the discovery of neutrophil extracellular traps (NETs), the extrusion of intracellular modified DNA and histones to trap microbes, as well as the highly proinflammatory neutrophil subset named low-density granulocytes (LDGs) during the last decade, interest in the neutrophil in SLE has seen a renaissance. However, there are data to suggest that neutrophils can play important roles in protection against, induction of and resolution of inflammation.

NETs were originally described as chromatin bound to granular and cytoplasmic molecules. However, today we know that the composition of NETs is highly dependent on the inducing stimulus and may contain characteristic lupus autoantigens such as DNA and chromatin. In SLE, LDGs spontaneously release NETs *ex vivo*, and *in vitro*, and autoantibodies directed against RNP induce release of neutrophil DNA able to subsequently mediate interferon (IFN)-alpha production by plasmacytoid dendritic cells (pDCs). Due to the suggested role of NETs in exposing autoantigens and supporting type I IFN production, several attempts have been made to inhibit NETosis in murine lupus models. So far, two main pathways - citrullination and oxidation - have been targeted. Whereas inhibition of the citrullination pathway (PAD4) reduced development of nephritis, deficiency in oxidation (Nox2) resulted in worsened lupus-like disease.

## Methods and results

We have studied the interplay between neutrophils and peripheral blood mononuclear cells (PBMCs) with regard to production of inflammatory mediators in response to nucleic acid-containing immune complexes (ICs). As demonstrated previously, RNA-containing ICs are efficient inducers of type I IFNs by either isolated pDCs or pDCs in PBMCs. However, when NETosis was induced by SLE ICs in the presence of PBMCs, production of type I IFNs, as well as other inflammatory cytokines, was markedly inhibited. This somewhat surprising finding may be explained by an inability of NETs to induce substantial inflammation in blood cultures due to the recently demonstrated anti-inflammatory effects through trapping and degradation of proinflammatory cytokines. In addition, neutrophils may compete for IC binding and internalization.

## Conclusions

We thus propose that in a physiological setting with the presence of both neutrophils and PBMCs, neutrophils may act as a sentinel capable of directing proinflammatory or anti-inflammatory responses depending on the presence of other cell types, spillage of NETs and other factors.

